# Facility factors influencing utilization of active management of third stage of labour among skilled birth attendants in Kiambu county, Kenya

**DOI:** 10.11604/pamj.supp.2016.25.2.9705

**Published:** 2016-11-26

**Authors:** Muiruri Felarmine, Osur Joachim, Okello Agina

**Affiliations:** 1 Amref Health Africa; 2 Kenyatta University, Nairobi City, Kenya

**Keywords:** Post-Partum Hemorrhage, Active Management of Third Stage of Labor, AMTSL, skilled birth attendants, health facility factors, maternal mortality

## Abstract

**Introduction:**

Post-Partum Hemorrhage (PPH) accounts for 34% of maternal deaths in Kenya. Active management of third stage of labour (AMTSL) is a World Health Organization and Ministry of Health of Kenya approved protocol for reducing maternal mortality and morbidity arising from post-partum hemorrhage. Kiambu County in Kenya records an average of six maternal deaths per month, out of which, two are due to PPH. This paper analyses how facility factors influence utilization of this protocol in Kiambu County.

**Methods:**

This was a cross sectional study among 431 skilled birth attendants in 52 health facilities. Two hundred and three birth attendants were selected using multistage sampling. Data was collected using questionnaires and observation checklists and analyzed using STATA version 11. Chi square, Fisher’s exact and Logistic regressions tests were used.

**Results:**

AMTSL was utilized by 31.5% of the birth attendants. Controlled cord traction (96.5%) was the most utilized. Uterine message after every 15 minutes was the least utilized component. Utilization was more in government facilities (37.4%) (Logistic regression p=0.006) and in level four health facilities (49.5%) (p<0.001). Utilization was higher (34.7%) among birth attendants who experienced less frequent stock outs (p=0.027) and in facilities with more than two staff authorized to order supplies (34.9%) (p=0.020). Utilization was higher in facilities with a fridge (44.5%) (p=0.001) and in facilities with standards documents in the labour ward (68.0%) (p=0.001).

**Conclusion:**

Health facility factors significantly influence utilization of AMTSL and therefore the county government should put in place strategies to enhance the factors that influence utilization of AMTSL positively.

## Introduction

Maternal mortality has remained high despite the fact that most maternal deaths are avoidable. About 1000 women die from pregnancy and childbirth related complications around the world every day. More than half of the deaths occur in sub-Saharan Africa and one third occur in South Asia [1]. World Health Organization (WHO) ranks Kenya as the eleventh country with the highest maternal mortality worldwide with a maternal mortality ratio (MMR) of 530 per 100,000 live births [1]. Kenya Demographic Health Survey (KDHS) reported MMR at 488 per 100,000 live births [2]. Despite the increased attention worldwide to reduce maternal mortality, in Kenya maternal mortality seems to have no significant change for the last 10 years; 414 per 100,000 in 2003 KDHS and 488 per 100,000 in 2008/2009 KDHS [2].

Looking at the main causes of the high maternal mortality, post-partum hemorrhage (PPH) which is bleeding after delivery tops the list accounting for 34% of maternal death [3]. PPH is also the most common complication of third stage of labour [4] affecting 14 million women per year of which 2% die [5]. Besides death, post PPH also causes serious morbidities such as respiratory distress syndrome, coagulopathy, shock, loss of fertility, pituitary necrosis and anemia. Many women who develop PPH require blood transfusion which sometimes can transmit blood borne infections such as Human Immunodeficiency Virus or cause adverse reactions [6]. Use of Active Management of Third Stage of Labour (AMTSL) by a skilled provider has been shown to decrease the incidence of PPH by up to 66% [3].

According to the Ministry of Health in Kenya guidelines, AMTSL involves three basic procedures: Administering oxytocin within one minute of child birth, delivering the placenta using controlled cord traction and massaging the uterus immediately after birth of the placenta and after every 15 minutes for the first 1-2 hours after delivery [7]. International health organizations such as WHO, International Federation of Gynecology and Obstetrics (FIGO), International Confederation of Midwives (ICM) as well as Ministry of Health in Kenya have endorsement use of AMTSL for safe delivery. This notwithstanding, the monthly health facility reports for the year 2011 in Kiambu County revealed that 6 women on average die per month and this translates to 2 women dying every month due to PPH.

Utilization of AMTSL is influenced by Health facility factors. This research article examines health facility factors influencing the utilization of AMSTL in Kiambu County in Kenya. According to KDHS 2008/2009, central province of Kenya, from which the county was mapped out, had most deliveries (73%) taking place in a health facility [2]. With such high health facility based deliveries, routine use of AMTSL can avert maternal deaths and morbidity due to PPH in this county. Knowledge of the health facility factors is important in designing of specific strategies to improve the utilization of AMTSL. This in turn will result in most mothers receiving AMTSL hence decrease incidence of postpartum hemorrhage.

## Methods

**Study design:** this study used cross-sectional design. This design described facility factors influencing utilization of AMTSL at the time of study.

**Study setting:** the study was carried out in Kiambu County located in central part of Kenya. In this county most mothers (74%) deliver in health facilities KDHS (2008/2009). The county has 378,574 women of reproductive age. The study was carried out in maternity units in the county. The county had a total of 52 health facilities offering routine delivery services in the county. These health facilities are owned by the either the government, faith based organizations or are privately owned. In terms of levels these health facilities included level three (health centers and nursing/midwifery homes) and level four facilities (hospitals).

**Study population:** the study population was skilled birth attendants working in maternity units in the 52 health facilities in Kiambu County. According to the districts health records and staff records in health facilities at the time of the study there were approximately 431 skilled birth attendants working in maternity units in the county.

**Sampling techniques and sample size:** multistage sampling was used. At the first stage, the birth attendants were stratified into six strata based on type (ownership) and the level of health facility. At second stage, the birth attendants in each stratum were clustered according to the health facility where they worked i.e. each health facility was regarded as a cluster. Simple random sampling using ballot method was used to sample the clusters included in the study from each stratum. Clusters were randomly selected until the proportionate number of birth attendants in the respective stratum was reached. A total of 28 clusters (health facilities) were sampled. All the birth attendants on duty (not on leave) during data collection in each of the 28 sampled clusters were interviewed. Each of the health worker sampled was observed managing third stage of labour and later interviewed. Therefore 203 birth attendants were interviewed and 203 deliveries observed. The Sample size was calculated using the Fisher’s formula when the population is less than 10,000.

**Data collection:** non-participant observation of management of third stage of labour was done using observation check list. Interviews using interviewer administered questionnaires were conducted for birth attendants who conducted the aforementioned deliveries to collect data on health facility factors. The research assistants were trained skilled birth attendants at diploma and degree levels.

**Variables:** the Independent variables were the health facility factors. The Dependent Variable was utilization of AMTSL. The care givers were grouped into two i.e. those who utilized and those who did not utilize. Utilization was determined by observing birth attendants conduct third stage of labour using an observation checklist. A care giver was said to have utilized AMTSL if he/she did all the following: Administered oxytocin within one minute of child birth, delivered the placenta using controlled cord traction, massaged the uterus immediately and massaged uterus after every 15 minutes for the first 1-2 hours after delivery. A care giver who did not perform one or more of the procedures was regarded not to have utilized. The observation check list however, collected information on performance of each of the procedures. This criterion was developed using the Ministry of Health guideline on components of AMTSL [7].

**Validity and reliability:** interviewer administered questionnaires were used rather than self-administered questionnaires to ensure validity. The questionnaires and the observation checklist were tested in a similar group and adjusted accordingly to improve their ability to collect appropriate data. To avoid hawthorne effect during observation of deliveries, the birth attendant were not informed of the specific skills being observed. Also observation of a birth attendant conducting third stage of labour was done before interviewing the birth attendant. The research assistants were qualified health workers who had been trained on AMTSL. The data collectors also underwent training on how to use the questionnaires and the checklists to ensure consistency.

**Data entry and analysis:** data was entered into STATA version 11. Descriptive statistics were used to generate frequencies and proportions. The dependent variable was organised as a binary variable with two categories; Utilized and not utilized AMTSL. Chi square and fisher’s exact at 95% confidence interval were used to test the association of the independent and dependent variables. The variables that had statistically significant association using chi square were subjected to logistic regression to generate the odds ratios.

## Results

### Demographic characteristics

Females were 81.8% of all respondents. Ages of the respondents were as follows; 32.0% aged between21-30, 35.0% aged between 31-40 years,25.1% aged between 41-50 years and 7.9% aged between 51-60 years.92.6% of the respondents were nurses and the others (8.4%) were clinical officers. 69.0 % of birth attendants were diploma holders, 27.6% were certificate holders and 3.4% were degree holders.

**Figure 1:**
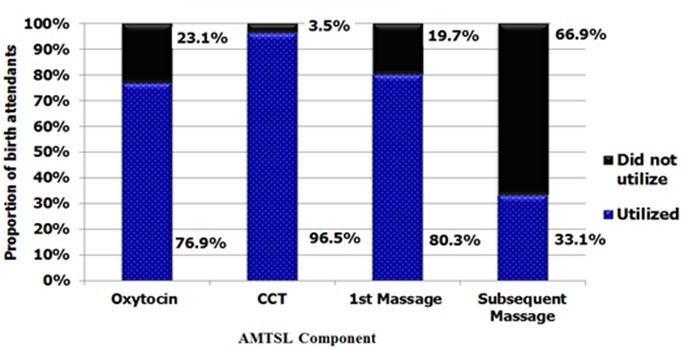
Extent of Utilization of individual components of active management of third stage of labour

AMTSL utilization status All (100%) the birth attendants utilized at least one of component of AMTSL as follows; 8.3% utilized one component, 27.0 % utilized two components, 33.0% utilized three components while 31.5% utilized all the four components of AMTSL. Controlled cord traction was the most utilized component with 96.5% of the care givers utilizing it. Uterine massage after every 15 minutes for 1-2 hours (subsequent massage) was the least utilized component with only 33.1% of birth attendants utilizing it. Figure 1 shows utilization of individual components of AMTSL.

### Type of health facility and utilization of AMTSL

**Figure 2:**
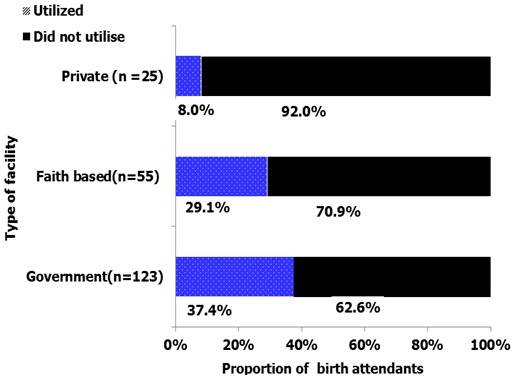
Association between type of health facility and utilization of active management of third stage of labour

The facilities type was determined using ownership (managing authority). The proportion of birth attendants utilizing AMTSL was highest among those working in government health facilities (37.4%) and was lowest was among those working in private health facilities (8.0%). The association between type of health facility and utilization of AMTSL was statistically significant at 95% confidence level (logistic regression p=0.006). Figure 2 shows association between type of health facility and utilization of AMTSL.

### Level of health facility and utilization of AMTSL

**Figure 3:**
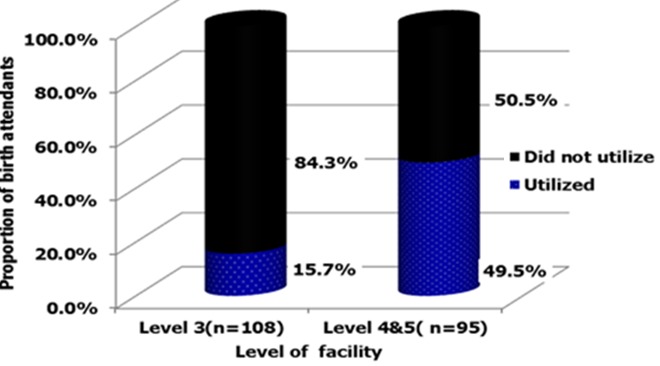
Association between level of facility and utilization of active management of third stage of labour

The level of health facility was determined using the Ministry of Health classification of health facilities (MOH, 2006). The proportion of birth attendants utilizing AMTSL was higher among birth attendants working in level 4 and 5 facilities at 49.5% than in level 3 facilities (15.7%). There was a statistical significant association between level of health facility and utilization of AMTSL at 95% confidence interval **(χ^2^= 26.6414, DF=1, P<0.001)**. Figure 3 shows Association between level of health facility and utilization of AMTSL.

### Supplies stock outs and utilization of AMTS

The birth attendants were requested to rate frequency of supply stock outs in a scale of one to five i.e. never, rarely, sometimes, very frequently and always. Ratings of never and rarely were regarded as ‘less frequent stock outs’ while ratings of sometimes, very frequently and always were regarded as ‘more frequent stock outs’. Supplies stock outs were less frequent for majority (83.7%) of the birth attendants. Birth attendants from faith based (2.9%) and government (7.6%) managed level four facilities are the least likely to have more frequent stock outs. Utilization of AMTSL was higher (34.7%) among birth attendants who had less frequent stock outs compared to care givers who experienced more frequent stock outs (15.1%). The association between supplies stock outs and utilization of AMTSL was statistically significant at 95% confidence interval **(χ^2^=4.8950, DF=1, p=0.027)**.

### Number of staff authorized to order supplies and utilization of AMTSL

Government managed facilities are more likely (93.5%) than other facilities to have more than two birth attendants authorized to order supplies. Most birth attendants (84.7%) worked in health facilities with more than 2 staff authorized to order supplies. Utilization of AMTSL was highest (34.9%) among birth attendants who worked in facilities with more than two staff authorized to order supplies. The association between number of staff authorized to order supplies and utilization of AMTSL was statistically significant at 95% confidence interval **(Fisher’s Exact p=0.020)**.

### Availability of supplies at the point of use and utilization of AMTSL

Availability of needles, syringes and oxytocin in labour ward was observed. The supplies were regarded as ‘not available’ if they were not available at the point of use (in labour ward) irrespective of their available in other places e.g. the store. Most (92.1%) birth attendants had supplies available at the point of use. Government level 4 facilities were more likely to have the supplies for AMTSL available at the point of use than other types of facilities. Utilization of AMTSL was higher (33.2%) among birth attendants working in facilities where supplies were available at the point of use than where supplies were not available at the point of use (12.5%). At 95% confidence interval, there was no statistically significant association between availability of supplies at the point of use and utilization of AMTSL **(Fisher’s Exact p= 0.100)**.

### Availability of a fridge and utilization of AMTSL

**Figure 4:**
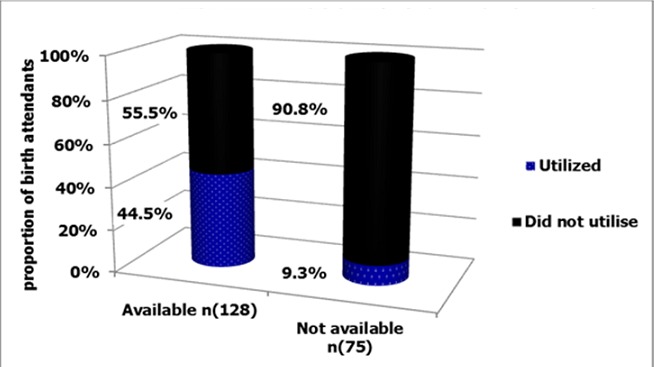
Association between availability of a fridge and utilization of active management of third stage of labour

Most (63.1%) of the birth attendants worked in facilities with a fridge. Faith based facilities (80.0%) and private facilities (72.0%) are more likely to have a fridge. On the other hand level 4 facilities (85.3%) are more likely than level 3 facilities to have a fridge (43.5%). Utilization of AMTSL was higher (44.5%) among birth attendants who worked in facilities with a fridge than those who worked in facilities without (9.3%). At 95% confidence interval, the association between availability of a fridge and utilization of AMTSL was statistically significant **(χ^2^=21.1398, DF=1, p=0.001)**. Figure 4 shows Association between availability of a fridge and utilization of AMTSL

### Availability of standard documents and utilization of AMTSL

**Figure 5:**
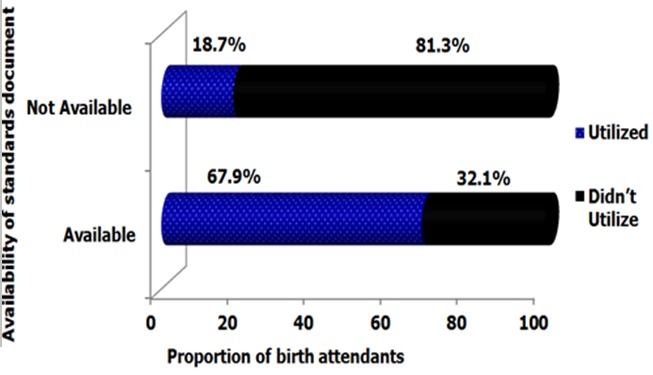
Association between availability of standards documents and active management of third stage of labour

Availability of standard documents in the maternity unit was observed. Absence of the documents in maternity units was termed as ‘not available’ irrespective of the presence of the documents elsewhere in the facility. Most (73.8%) birth attendants worked in health facilities with no AMTSL standards documents. Birth attendants working in government level 4 facilities are the most likely to have AMTSL standards documents in the labour ward (83%) than others. Utilization of AMTSL was highest (68.0%) among birth attendants who worked in facilities with standards documents in the labour ward and the lowest (16.6%) among birth attendants with no standards documents. The association between availability of standards documents and utilization of AMTSL was statistically significant at 95% confidence interval **(χ²=44.017, DF=1, p=0.001)**. Figure 5 shows the Association between availability of AMTSL standards documents and utilization of AMTSL.

Logistic regression of facility factors at 95% confidence interval Working in private hospitals reduced the likely hood of utilizing AMTSL 0.5 times. Working in level 4 health facilities increased likely hood of utilizing AMTSL 5.2 times. Having more than two staff authorised to order supplies increased the likely hood of utilizing AMTSL 3.6 times. Having a fridge at health increased the likely hood of utilizing AMTSL 7.8 times. Availability of standards document in the labour ward increased the likely hood of utilizing AMTSL 3 times. Table 1 shows the logistic regression of facility factors at 95% confidence interval.

**Table 1:** Logistic regression of the facility factors

Variable	Odds Ratio	P>│z│
Type of facility	0.502^+^	0.006
Level of facility	5.241^+^	<0.001
Stock outs	2.017	0.147
Authority to order supplies	3.616^+^	0.022
Availability of a fridge	7.799^+^	<0.001
Availability of standards documents in the labour ward	3.037^+^	<0.001

## Discussion

Most of the birth attendants in Kiambu County do not utilize AMTSL as per the guidelines. In neighboring countries like Tanzania and Ethiopia the utilization of AMTSL is even lower with only (7%) and (4.5%) of the deliveries respectively benefiting from correct use of AMTSL [8].

Oxytocin administration and Controlled cord traction were the most utilized components of AMTSL. This can be explained by the fact that these two components were in the former guidelines of management of third stage of labour. The former guidelines recommended oxytocin to be administered at the birth of anterior shoulder or immediately after the birth of the baby. The current guidelines recommend oxytocin administration strictly within a minute of child birth. This means that birth attendants who followed the second option of the former guideline were correct in regard to the current guideline.

Oxytocin administration and controlled cord traction also take less time to perform and they are one off time procedures unlike uterine message after every 15 minutes for 2hrs which was the least adhered to component. A qualitative study done to assess factors influencing nurses compliance to standard procedures identified ‘time consuming procedures’ as reasons behind non-compliance [9].

There was statistically significant association between both type and level of health facility and utilization of AMTSL in Kiambu County. There was more utilization of AMTSL in Government and level 4 facilities. This discrepancy can be attributed to factors such as availability of standards documents in labour, authorization to order supplies and birth attendance factors such as staff training, knowledge on AMTSL and cadre of the birth attendants. These factors were more favorable in Government and level 4 facilities.

AMTSL supplies (needles, syringes) are available at the point of use for most of the birth attendants in Kiambu County. This relates well with the finding of this study that stock outs for these supplies were not frequent for majority of birth attendants. Other studies in Kenya have reported similar findings for instance a national survey reported that 71% of health facilities in Kenya have oxytocin and 96% of the health facilities have syringes and needles within reach in delivery room [10]. In Tanzania the situation is not different as oxytocin was reported to be available in 97% of the health facilities [8]. Outside Africa supplies for AMTSL are fairy adequate for instance in Guatemala 93.3% of health facilities have been reported to have oxytocin [11].

The findings of this study that level 4 and 5 facilities are more likely to have AMTSL supplies is similar to a national survey that reported that availability of medicines and supplies for delivery are more likely to be available in hospitals (level 4 and 5) (72 %) [10]. On the other hand, the findings that government facilities are more likely to have AMTSL supplies than private and FBO facilities differs from the national survey that rated private and FBO facilities (68%) better in terms of availability of medicine and other delivery supplies than government facilities. The discrepancy may be due to the fact that the survey assessed availability of all essential supplies for delivery including drugs but this study only assessed availability of needles, syringes and oxytocin. In addition this study assessed the availability of the supplies at the point of use i.e. in the labour room unlike the survey that assessed the availability of supplies within the health facility and not necessarily in labour room.

Fridges are available in most facilities in Kiambu County. Fridges are important for proper storage of oxytocin. The statistically significant association between availability of a fridge and higher utilization of AMTSL can be explained by findings of a qualitative study done on compliance with standard precautions. This study found out that availability of equipment or storage of such equipment in places far from where care is provided is a barrier to provision of nursing care [9].

In Kiambu county, faith based and private managed facilities are more likely to have a fridge than government facilities. Also Level 4 facilities are more likely than level 3 facilities to have a fridge. This relates well to the findings by the national survey that hospitals, private and faith based facilities are more likely to have power supply than other facilities.

Availability of standards documents in health facilities in Kiambu County is low. Other studies have had similar finding, a national survey reported a 27% availability of obstetric care (not AMTSL) standards documents in central province [10]. However, in other countries like Guatemala standards documents are more available in facilities (66.7 %) AMTSL [11]. The association between availability of standards document and utilization of AMTSL is statistically significant.

Standards documents are more available in level 4 and government facilities compared to other facilities. Kenya Service Provision Assessment Survey also reported higher availability of standards documents in government managed and level 4 facilities. This is likely to be due to the fact that standards documents are generated by the government and this means they are likely to be distributed to government facilities before others.

This study did not examine the outcome of the utilization of AMTSL. There is therefore need for further research to find out the outcome of utilization of AMTSL. This study also covered only one county and therefore the results could not be generalized to the whole country, national wide study is therefore necessary.

## Conclusion

Utilizations of AMTSL was low. Utilization of AMTSL was higher in government health facilities and level 4 health facilities. Factors such as less frequent supplies stock outs, having more than two staff authorized to order supplies, having a fridge and having AMTSL standards documents in the labour ward. The county government and stakeholders in health care should put strategies to accelerate utilization of AMTSL by ensuring that facilitating factors are in place and more so in level three and private health facilities.

### What is known about this topic

- The effectiveness of Active Management of Third stage of labour in Kenya;
- Utilization of Active Management of Third stage of labour in Kenya in a few African countries and in Latin America.

### What this study adds

- Information on adherence to active management of third stage of labour guidelines in Kiambu county, Kenya;
- Information on factors influencing utilization of Active Management of Third stage of labour in Kenya.
